# ^99m^Tc-3P_4_-RGD_2_ Scintimammography in the Assessment of Breast Lesions: Comparative Study with ^99m^Tc-MIBI

**DOI:** 10.1371/journal.pone.0108349

**Published:** 2014-09-24

**Authors:** Qingjie Ma, Bin Chen, Shi Gao, Tiefeng Ji, Qiang Wen, Yan Song, Lei Zhu, Zheli Xu, Lin Liu

**Affiliations:** 1 China-Japan Union Hospital, Jilin University, Changchun, China; 2 State Key Laboratory of Molecular Vaccinology and Molecular Diagnostics & Center for Molecular Imaging and Translational Medicine, School of Public Health, Xiamen University, Xiamen, China; Queensland Institute of Medical Research, Australia

## Abstract

**Purpose:**

To compare the potential application of ^99m^Tc-3P-Arg-Gly-Asp (^99m^Tc-3P_4_-RGD_2_) scintimammography (SMM) and ^99m^Tc-methoxyisobutylisonitrile (^99m^Tc-MIBI) SMM for the differentiation of malignant from benign breast lesions.

**Method:**

Thirty-six patients with breast masses on physical examination and/or suspicious mammography results that required fine needle aspiration cytology biopsy (FNAB) were included in the study. ^99m^Tc-3P_4_-RGD_2_ and ^99m^Tc-MIBI SMM were performed with single photon emission computed tomography (SPECT) at 60 min and 20 min respectively after intravenous injection of 738±86 MBq radiotracers on a separate day. Images were evaluated by the tumor to non-tumor localization ratios (T/NT). Receiver operating characteristic (ROC) curve analysis was performed on each radiotracer to calculate the cut-off values of quantitative indices and to compare the diagnostic performance for the ability to differentiate malignant from benign diseases.

**Results:**

The mean T/NT ratio of ^99m^Tc-3P_4_-RGD_2_ in malignant lesions was significantly higher than that in benign lesions (3.54±1.51 vs. 1.83±0.98, p<0.001). The sensitivity, specificity, and accuracy of ^99m^Tc-3P_4_-RGD_2_ SMM were 89.3%, 90.9% and 89.7%, respectively, with a T/NT cut-off value of 2.40. The mean T/NT ratio of ^99m^Tc-MIBI in malignant lesions was also significantly higher than that in benign lesions (2.86±0.99 vs. 1.51±0.61, p<0.001). The sensitivity, specificity and accuracy of ^99m^Tc-MIBI SMM were 87.5%, 72.7% and 82.1%, respectively, with a T/NT cut-off value of 1.45. According to the ROC analysis, the area under the curve for ^99m^Tc-3P_4_-RGD_2_ SMM (area = 0.851) was higher than that for ^99m^Tc-MIBI SMM (area = 0.781), but the statistical difference was not significant.

**Conclusion:**

^99m^Tc-3P_4_-RGD_2_ SMM does not provide any significant advantage over the established ^99m^Tc-MIBI SMM for the detection of primary breast cancer. The T/NT ratio of ^99m^Tc-3P_4_-RGD_2_ SMM was significantly higher than that of ^99m^Tc-MIBI SMM. Both tracers could offer an alternative method for elucidating non-diagnostic mammograms.

## Introduction

Breast cancer continues to be a major public health problem all over the world. The American Cancer Society estimates that there will be about 296,980 new cases of breast cancer in 2013, which is expected to account for 14% of female cancer deaths.

A realistic strategy for the reduction of breast cancer mortality rates and timely treatment is to detect the disease while it is still in an early stage.[1], [2]. The most common screening method for early breast cancer is mammography, which is very sensitive in the detection of malignant breast disease. However in several groups of breast cancer patients, including those with fibroadenoma breasts, post implants, mastectomy or severe dysplasia, mammography has a low predictive value (20%–30%) and is not accurate, requiring patients to undergo histopathological examinations for a definitive diagnosis [3], [4]. To improve diagnostic accuracy, new methods are being studied as alternatives to mammography. Over the last twenty years, Scintimammography (SMM) has been introduced as an adjunct modality to present imaging modalities for breast cancer imaging [5]. In addition to the imaging modality, several radiopharmaceuticals have also been investigated for diagnostic imaging procedures in patients with suspected breast cancer [6]. ^18^F-fluorodeoxyglucose (FDG) positron emission tomography (PET) [7] is proven to be the most effective in detection of breast cancer for diagnosis, staging and restaging, but its use is limited by the high cost of equipment and lack of general availability, especially in developing countries. Alternatively, single photon emission computed tomography (SPECT) is more widely used with a much lower cost worldwide.

^99m^Tc-methoxyisobutylisonitrile (^99m^Tc-MIBI) is an important tracer for oncological applications and has been widely used in breast tumor imaging. However, this tracer originated from nuclear medicine for cardiac imaging and was not specifically designed for tumor imaging. The exact mechanism of uptake in breast cancer cells is still not entirely clear. It is reported that ^99m^Tc-MIBI is concentrated in cancer cells by an energy-requiring transport mechanism, specifically by transmembrane electrical potentials, as well as by non-specific mechanisms, and the tracer is stored within the mitochondria [8].

It is well documented that integrin αvβ3 plays a critical role in the regulation of tumor angiogenesis and metastasis [9], [10]. The integrin is upregulated on activated endothelial cells and is highly expressed in tumor cells of various tumor types, including breast cancer [11], [12]. Over the past decade, radiolabeled Arg-Gly-Asp (RGD) peptides and analogs that specifically target integrin αvβ3 have been intensively investigated for noninvasive imaging of tumors in pre-clinical and clinical studies [13]–[19]. We previously developed the αvβ3-specific tracer ^99m^Tc-3P-Arg-Gly-Asp (^99m^Tc-3P_4_-RGD_2_) for SPECT and already demonstrated that ^99m^Tc-3P_4_-RGD_2_ SPECT allows specific imaging of αvβ3 expression with high accuracy in detecting malignant solitary pulmonary nodules (SPNs), esophageal cancer, and malignant gliomas [20]–[22].

In this study, we compare the diagnostic value of ^99m^Tc-3P_4_-RGD_2_ SMM with ^99m^Tc-MIBI SMM for the detection of breast cancer by receiver operating characteristic (ROC) curve analysis.

## Materials and Methods

### Patients

Thirty-six patients with breast masses on physical examination and/or suspicious mammographic findings that required fine needle aspiration cytology biopsy (FNAB) were included in this study. The patient mean age was 41.9±12.2 years (age range 22–65 years. All patients were referred for ^99m^Tc-MIBI and ^99m^Tc-3P_4_-RGD_2_ SMM on an individual basis. The time interval between the two imaging procedures was 3.2±1.4 days. Finally, ^99m^Tc-3P_4_-RGD_2_ and ^99m^Tc-MIBI SMM results were compared with each other and with the final histopathological diagnosis. Inclusion and exclusion criteria for entry into the study are summarized in Table 1. This study was approved by the Ethics Committee of China-Japan Union Hospital of Jilin University. Informed written consent to participate in the SMM studies was obtained from all patients.

**Table 1:** Inclusion and exclusion criteria of study.

Inclusion Criteria	Exclusion Criteria
Female	Pregnancy
Not pregnant	Recurrent disease
Suspicious lesion of the breast	Pervious mastectomy
Recommendation for excision biopsy after mammography	Fine needle aspiration within 1 week prior to scintimammography
Informed consent from the patient	Previous chemotherapy
	Medically unstable patient (severe arrhythmia, heart failure or recent surgery)

### Scintimammography protocol

Radiolabeling and quality control procedures for 3P_4_-RGD_2_ were performed as described previously [20]. Both 3P_4_-RGD_2_ and MIBI (ShiHong Drug Development Center, Beijing, China) were radiolabelled with 738±86 MBq ^99m^technetium and thereafter administered via a single intravenous bolus injection in the contralateral arm to the affected breast, followed by a 10 mL saline flush. The effective radiation dose to the body of ^99m^Tc-3P_4_-RGD_2_ and ^99m^Tc-MIBI were 2.89±0.34 mSv and 5.83±0.67 mSv, respectively [23], [24]. ^99m^Tc-3P_4_-RGD_2_ and ^99m^Tc-MIBI SMM were performed at 60 min and 20 min after intravenous injection, respectively. Patients were in supine position with raised arms during imaging.

SPECT was performed using a double-head γ camera (Precedence, Philips Healthcare), equipped with low-energy parallel hole collimators. The matrix was 128×128 pixels, and the photopeak was centered at 140 keV with a symmetrical 20% window. Imaging with both radiotracers was performed using 6° angular steps in a 20 s time frame. Distance between the breast and detector was minimized.

### Data analysis

Both ^99m^Tc-3P_4_-RGD_2_ and ^99m^Tc-MIBI SMM uptake were evaluated by semiquantitative analysis. Regions of interest (ROIs) were drawn around the tumor and an area of normal breast tissue in the same breast on lateral images and used to determine the tumor to non-tumor ratios (T/NT) of ^99m^Tc-3P_4_-RGD_2_ and ^99m^Tc-MIBI.

All numerical results are reported as mean values with standard deviations (SDs). Student's t test was used for statistical comparison of quantitative indices between the malignant and benign breast disease groups. The IBM SPSS Statistics19 software was used to determine cut-off values of quantitative indices in the detection of primary breast cancer. The incremental diagnostic value of quantitative indices analysis was performed using calculated areas under the curve (AUCs) in ROC analysis. Statistical significance was defined as p<0.05.

## Results

Samples for histological examination were obtained by surgery in 28 patients and by core needle biopsy in eight patients. Breast cancer was confirmed in 26 patients and resulted in a total of 28 cancer lesions with diameters ranging from 0.3 cm to 7.9 cm (mean ± SD: 2.86±1.73 cm). Benign breast disease was found in 10 patients with a total of 11 benign lesions ranging in diameter from 0.4 cm to 6.5 cm (mean ± SD: 2.83±1.91 cm). In this study, the yielding breast cancer prevalence was 71.8% (Table 2).

**Table 2:** Scintimammography results versus final histopathological diagnosis of 36 patients.

Patient	Age (years)	Diameter (cm)	RGD (T/NT)	MIBI (T/NT)	Histopathological Diagnosis
1	58	2.8	4.55	2.70	Invasive ductal
2	43	4.2	2.81	1.90	Invasive lobular
3	26	0.6	1.31	1.02	Invasive ductal
4	52	3.2	3.51	1.70	DCIS
5	53	2.5	5.70	3.10	Invasive ductal
6	65	1.8	3.33	1.90	Invasive ductal
7	45	0.9	2.71	1.54	Invasive ductal
8	59	7.9/3.0	4.24/3.32	3.51/1.85	Invasive ductal/Invasive ductal
9	49	0.3	1.29	1.14	Invasive ductal
10	49	1.8	2.91	1.68	Invasive ductal
11	33	3.7	2.48	2.33	Invasive ductal
12	23	2.5	5.04	3.65	Invasive ductal
13	36	0.4	1.02	1.23	Invasive ductal
14	29	6.0	8.27	4.87	Invasive lobular
15	31	2.2	2.96	1.31	DCIS
16	56	4.2	3.82	2.08	Invasive ductal
17	41	3.5	5.62	4.21	Invasive mucinous
18	37	3.8	4.20	2.10	Invasive ductal
19	22	4.5	2.52	1.62	Invasive ductal
20	31	1.7/0.8	4.12/2.49	1.85/1.91	Invasive ductal/DCIS
21	39	4.1	3.34	2.17	Invasive ductal
22	61	1.2	3.40	3.74	Invasive ductal
23	46	3.3	2.99	1.61	Invasive mucinous
24	58	2.9	5.23	3.21	Invasive ductal
25	27	2.0	3.01	1.60	Invasive lobular
26	44	4.2	2.79	1.82	Invasive ductal
27	41	4.3	1.11	1.34	Fibroadenoma
28	28	2.1	4.47	2.58	Fibroadenoma with mastitis
29	31	6.5/0.7	1.92/1.32	1.26/1.10	Fibroadenoma/ductal ectasia
30	47	3.2	1.34	2.81	Fibroadenoma
31	29	1.8	1.43	1.37	Fibrocystic disease
32	54	5.2	2.31	1.22	Fibroadenoma
33	49	3.3	1.1	1.09	Fibroadenoma
34	37	1.2	1.85	1.69	Fibroadenoma
35	25	2.4	2.17	1.02	Fibrocystic disease
36	55	0.4	1.08	1.17	Ductal ectasia

DCIS: ductal carcinoma in situ.

**Figure 1:**
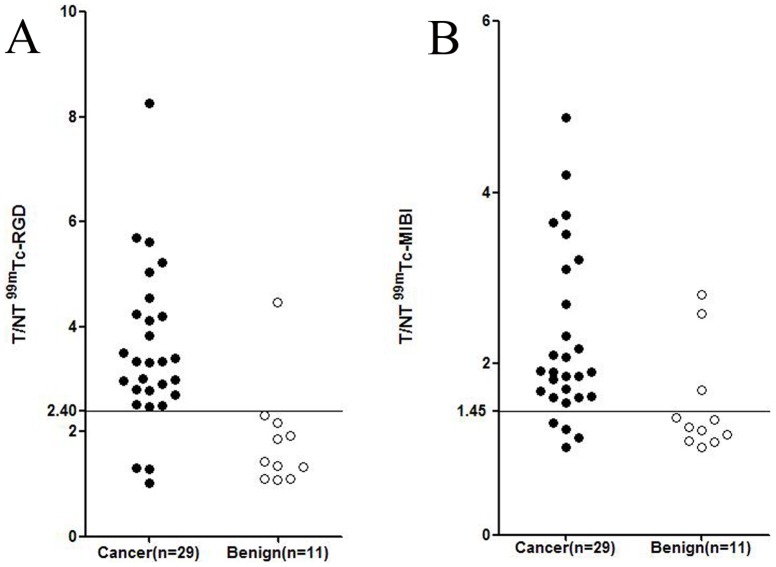
T/NT for ^99m^Tc-RGD and ^99m^Tc-MIBI in malignant and benign tumors. (A) The T/NT for ^99m^Tc-RGD in breast cancer was significantly higher than that in benign lesions (p<0.001). (B) The T/NT for ^99m^Tc-MIBI in breast cancer was significantly higher than that in benign lesions (p<0.001).

^99m^Tc-3P_4_-RGD_2_ SMM was false negative in 3 breast cancer of invasive ductal which was the same as ^99m^Tc-MIBI SMM. The tumor size was 0.6 cm or smaller in the long axis diameter. One patient with ductal carcinoma in situ (DCIS) in the long axis diameter of 2.2 cm was clear detected by ^99m^Tc-3P_4_-RGD_2_ SMM, but not with ^99m^Tc-MIBI SMM (Fig. 2). ^99m^Tc-MIBI SMM was false positive in 3 benign lesions. Of the false positive cases, two were fibroadenoma and one was fibroadenoma with mastitis. The fibroadenoma with mastitis was also false positive in ^99m^Tc-3P_4_-RGD_2_ SMM (Fig. 3).

We observed high ^99m^Tc-3P_4_-RGD_2_ uptake in breast cancer and low ^99m^Tc-3P_4_-RGD_2_ uptake in benign lesions (Fig. 1A). In ^99m^Tc-3P_4_-RGD_2_ SMM, the T/NT of breast cancer was 3.54±1.51 and that of benign lesions was 1.83±0.98. The difference was statistically significant (p<0.001). Similarly in ^99m^Tc-MIBI SMM, high MIBI uptake was observed in breast cancer while low MIBI uptake was detected in benign lesions (Fig. 1B). The T/NT of breast cancer was 2.86±0.99 and that of benign lesions was 1.51±0.61. The difference was statistically significant (p<0.001).

**Figure 2:**
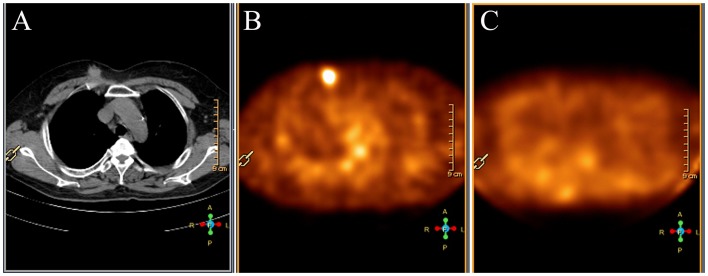
A 2.2 cm ductal carcinoma in situ of the right breast in a 31-year-old woman (Patient 15). (A) CT scan demonstrates a mass in the right breast. (B) ^99m^Tc-3P_4_-RGD_2_ SMM demonstrates focal uptake of ^99m^Tc-3P_4_-RGD_2_ in the tumor (T/NT = 2.96). (C) ^99m^Tc-MIBI SMM demonstrates low uptake of ^99m^Tc-MIBI in the tumor (T/NT = 1.31).

**Figure 3:**
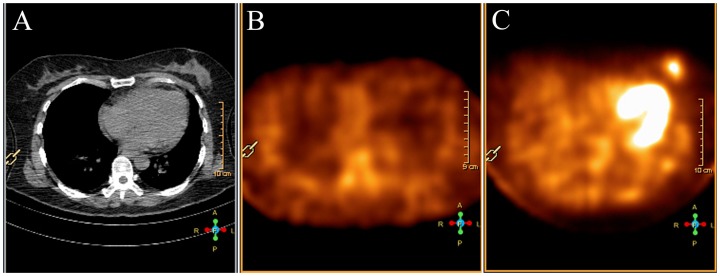
A 3.2 cm fibroadenoma of the left breast in a 47-year-old woman (Patient 30). (A) CT scan demonstrates a mass in the left breast. (B) ^99m^Tc-3P_4_-RGD_2_ SMM demonstrates low uptake of ^99m^Tc-3P_4_-RGD_2_ in the tumor (T/NT = 1.34). (C) ^99m^Tc-MIBI SMM demonstrates focal uptake of ^99m^Tc-MIBI in the tumor (T/NT = 2.81).

**Figure 4:**
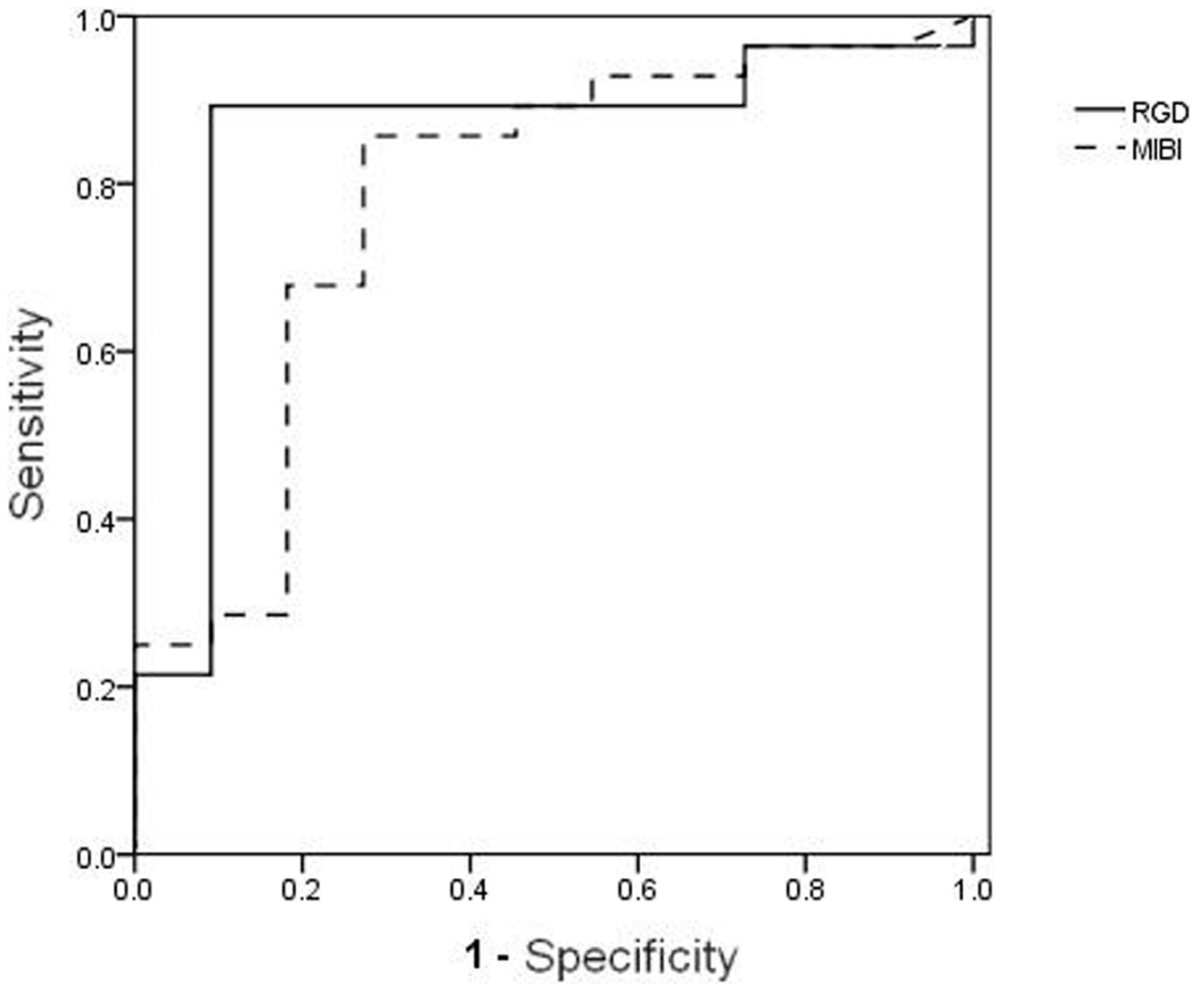
Comparison the sensitivity and specificity of ^99m^Tc-3P_4_-RGD_2_ SMM and ^99m^Tc-MIBI SMM. Comparison between ^99m^Tc-3P_4_-RGD_2_ SMM and ^99m^Tc-MIBI SMM in the differential diagnosis of breast cancer and benign lesions using ROC analysis (solid line: ^99m^Tc-3P_4_-RGD_2_ SMM, dashed line: ^99m^Tc-MIBI SMM). The area under the curve of both ^99m^Tc-3P_4_-RGD_2_ SMM and ^99m^Tc-MIBI SMM are 0.851 and 0.781, respectively. The difference was not significant.

ROC analyses were performed to determine the optimal cut-off values of both ^99m^Tc-3P_4_-RGD_2_ and ^99m^Tc-MIBI SMM T/NT for the detection of malignant breast cancer. When a cut-off value was used based on the ROC analysis, the sensitivity, specificity and accuracy of ^99m^Tc-3P_4_-RGD_2_ SMM were 89.3%, 90.9% and 89.7%, respectively (cutoff = 2.40 of T/NT), and those of ^99m^Tc-MIBI SMM were 87.5%, 72.7% and 82.1%, respectively (cutoff = 1.46 of T/NT). The empirical ROC areas, which estimate the overall diagnostic performance, did not differ significantly among the two diagnostic analyses (Fig. 4). The value was 0.851 for ^99m^Tc-3P_4_-RGD_2_ SMM and 0.781 for ^99m^Tc-MIBI SMM.

## Discussion

Over the last twenty years, SMM has been proposed to be a complementary tool to mammography in the diagnosis of primary breast cancer [5]. An already widely used radiopharmaceutical, ^99m^Tc-MIBI appears to be a suitable SMM scanning agent. Many publications have reported favorable sensitivity and specificity results, 84%–96% and 72%–94%, respectively, for ^99m^Tc-MIBI scintigraphy in the diagnosis of breast cancer [25]–[32]. ^99m^Tc-3P_4_-RGD_2_ is a new agent with a high affinity for the αvβ3 integrin, a receptor associated with angiogenesis. In our previous study, we found that ^99m^Tc-3P_4_-RGD_2_ could accumulate in a variety of malignant lesions [20]–[22]. However, a comparative study between ^99m^Tc-3P_4_-RGD_2_ and ^99m^Tc-MIBI SMM has not been previously reported.

In this present study, to differentiate benign from malignant lesions, ROC analyses were performed to determine the optimal cut-off values of T/NT of ^99m^Tc-3P_4_-RGD_2_ and ^99m^Tc-MIBI SMM. When T/NT of 2.40 was used as a cut-off point, the sensitivity, specificity and accuracy of ^99m^Tc-3P_4_-RGD_2_ SMM were 89.3%, 90.9% and 89.7%, respectively. With a T/NT of 1.45 as a cut-off value, the same findings were 87.5%, 72.7% and 82.1% in ^99m^Tc-MIBI SMM, respectively. The sensitivities reported in this study for ^99m^Tc-3P_4_-RGD_2_ SMM are comparable with our previous reports; however the specificity is slightly higher than previous studies, which may be due to the low total number of benign breast lesions [20]–[22]. For ^99m^Tc-MIBI SMM, the results reported here are comparable with those in previous studies [25]–[32]. Although the sensitivity, specificity and accuracy of ^99m^Tc-3P_4_-RGD_2_ SMM was slightly superior to that of ^99m^Tc-MIBI SMM in this study, the difference was not statistically significant. The area under the curve of ^99m^Tc-3P_4_-RGD_2_ SMM was slightly larger than that of ^99m^Tc-MIBI SMM, although this difference was also not significant.

It is generally accepted that the detection sensitivity of SMM is much lower for small breast cancer lesions with a diameter less than 1 cm over larger lesions [29]. Data from a multicentre European study showed a sensitivity of 26%–56% for lesions less than 1 cm [33]. Similarly in the present study, neither ^99m^Tc-3P_4_-RGD_2_ nor ^99m^Tc-MIBI was sufficient to visualize tumors in three patients having malignant lesions with diameters at 0.3 cm, 0.4 cm and 0.6 cm, respectively. The ^99m^Tc-3P_4_-RGD_2_ and ^99m^Tc-MIBI uptake in small lesions is considered to be underestimated due to partial volume effects from the relatively low spatial resolution of the SPECT device, though other factors such as the degree of radiopharmaceutical uptake by tumors and normal breast tissue may have contributed as well.

In one case of DCIS with a long axis diameter of 2.2 cm was false negative on ^99m^Tc-MIBI SMM but not ^99m^Tc-3P_4_-RGD_2_ SMM. Some reports state that ^99m^Tc-MIBI SMM always showed a low sensitivity for detecting DCIS. Vassilios Papantoniou et al. [34] studied the diagnostic accuracy of ^99m^Tc-MIBI SMM in 13 cases of DCIS and achieved a low sensitivity of 46%. Reinhard Obwegeser et al. [35] reported that ^99m^Tc-MIBI SMM could not detect all four DCIS in their study. They conceived it may be due to the histological type of DCIS, which is known to show a lower density of tumor cells per square unit than invasive ductal carcinomas. Conversely, experimental studies using in vivo assays have shown that breast carcinoma in situ may be antigenic [36]. Sections stained for endothelial markers have shown increased vascularity around DCIS [37]–[39]. A more detailed study demonstrated two patterns of increased vascularity: cuffs of vessels close to the involved ducts and vessels diffusely arranged in the interductal stroma [40]. The true positive result with ^99m^Tc-3P_4_-RGD_2_ SMM in this common malignant tumor may be an advantage of RGD targeting. Further studies with a larger patient population is needed to determine this issue.

Three of the 11 patients with fibroadenoma showed focal ^99m^Tc-MIBI uptake, and one patient from this group who was diagnosed with fibroadenoma with severe mammitis on histopathological examination showed high focal tracer accumulation of ^99m^Tc-3P_4_-RGD_2_. The false positive results obtained with ^99m^Tc-MIBI in three fibroadenoma may be due to the high cellular activity associated in fibroadenoma. Previous studies have demonstrated that integrin αvβ3 is preferentially expressed on several types of cancer cells including melanoma, glioma, and ovarian and breast cancer. However, because expression is very low in existing blood vessels and absent in normal tissue, the accumulation of ^99m^Tc-3P_4_-RGD_2_ may be mainly due to its higher specificity [41]–[44]. As is known to all, inflammation was different from other benign lesions, always showed high cell density and vascularity, likely responsible for the increased uptake. Previous studies have also shown that the integrin αvβ3 can exist on neutrophils, monocytes, and vascular smooth muscle cells [45], which can be the main reason for the false positive result using ^99m^Tc-3P_4_-RGD_2_ SMM.

In conclusion, ^99m^Tc-3P_4_-RGD_2_ SMM does not provide any significant advantage over the established ^99m^Tc-MIBI SMM for differentiating breast lesions. The uptake of ^99m^Tc-3P_4_-RGD_2_ in breast cancer was higher than that of ^99m^Tc-MIBI. ^99m^Tc-3P_4_-RGD_2_ seems to be more accurate than ^99m^Tc-MIBI in the detection of DCIS and fibroadenoma. But with only a few patients, there was no statistically significant difference between ^99m^Tc-3P_4_-RGD_2_ and ^99m^Tc-MIBI SMM. Future studies will involve higher sample numbers.
